# CRISPR/Cas12a Technology Combined with Recombinase Polymerase Amplification for Rapid and Portable Monkeypox Virus Detection

**DOI:** 10.1128/spectrum.04233-22

**Published:** 2023-05-08

**Authors:** Feifei Li, Sihua Liu, Boyu Luo, Mengqian Huang, Yue Teng, Tao Wang

**Affiliations:** a School of Life Sciences, Tianjin University, Tianjin, China; b State Key Laboratory of Pathogen and Biosecurity, Beijing Institute of Microbiology and Epidemiology, Beijing, China; Chinese Academy of Sciences Wuhan Institute of Virology

**Keywords:** monkeypox virus, CRISPR/Cas12a, RPA, one-pot reaction, detection

## LETTER

Monkeypox virus (MPXV) continues to circulate in several countries in which it is not endemic, posing a potential threat to human health and world stability (1). Here, we developed a CRISPR/Cas12a approach combined with recombinase polymerase amplification (RPA) for MPXV testing within 40 min. Importantly, the two reaction systems were further integrated into a one-step fluorescence assay by optimizing the protospacer adjacent motif (PAM) sequence. The whole detection process can be completed within 30 min.

**FIG 1:**
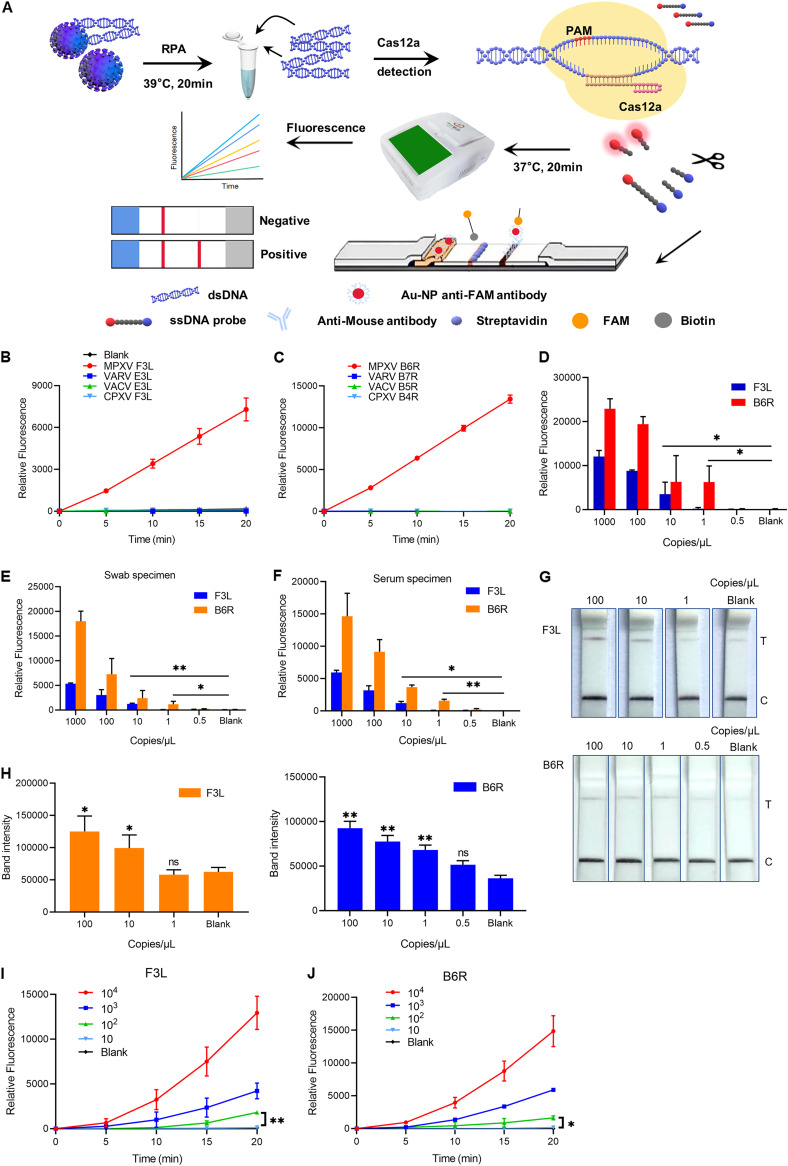
CRISPR/Cas12a technology combined with RPA for MPXV detection. (A) Schematic diagram of Cas12a combined with RPA for MPXV detection. The MPXV F3L and B6R genes were amplified by RPA at 39°C for 20 min. The Cas12a system was then added to identify and combine with dsDNA under the guidance of the crRNA. Cas12a randomly cleaved the ssDNA reporters (see Table S1), and then the fluorescence signal could be captured by the GS8 isothermal cycler or immunochromatographic strips. (B) DNA of MPXV (final concentration: F3L gene, 100 copies/μL), VARV, VACV, and CPXV amplified by RPA and detected by the CRISPR/Cas12a system. (C) DNA of MPXV (final concentration: B6R gene, 100 copies/μL), VARV, VACV, and CPXV amplified by RPA and detected by the CRISPR/Cas12a system. (D) Histogram showing the sensitivity of CRISPR/Cas12a combined with RPA for detection of the F3L and B6R genes at 20 min. A serially diluted synthetic MPXV plasmid was used as the template. The unpaired *t* test was applied for the statistical analysis in GraphPad Prism v8. *, *P* < 0.05. (E) Histogram showing the sensitivity of CRISPR/Cas12a combined with RPA for detection of the F3L and B6R genes at 20 min. The sensitivity of the system for the simulated specimen was tested after gradient dilution of DNA extracted from a simulated swab sample. The unpaired *t* test was applied for the statistical analysis in GraphPad Prism v8. *, *P* < 0.05; **, *P* < 0.01. (F) Histogram showing the sensitivity of CRISPR/Cas12a combined with RPA for detection of the F3L and B6R genes at 20 min. The sensitivity of the system for the simulated sample was tested after gradient dilution of DNA extracted from a simulated serum sample. The unpaired *t* test was applied for the statistical analysis in GraphPad Prism v8. *, *P* < 0.05; **, *P* < 0.01. (G) Sensitivity of CRISPR/Cas12a combined with lateral flow strips for detection of the F3L and B6R genes. Serially diluted synthetic MPXV DNA (final concentration: F3L, 100 to 0 copies/μL; B6R, 100 to 0 copies/μL) was used as the template. T, test; C, control. (H) Visualization of the sample test band intensity quantified by ImageJ and GraphPad. The unpaired *t* test was applied for the statistical analysis in GraphPad Prism v8. *, *P* < 0.05; **, *P* < 0.01; not significant [ns], *P* > 0.05. (I) Sensitivity of the one-pot test using TTGG crRNA targeting the F3L gene. Serially diluted synthetic MPXV DNA (final concentration: F3L, 10^4^ to 10 copies/μL) was used as the template. Diethyl pyrocarbonate (DEPC)-treated water was used as the template for the blank group. The unpaired *t* test was applied for the statistical analysis in GraphPad Prism v8. **, *P* < 0.01. (J) Sensitivity of the one-pot test using TTGG crRNA targeting the B6R gene. Serially diluted synthetic MPXV DNA (final concentration: B6R, 10^4^ to 10 copies/μL) was used as the template. DEPC-treated water was used as the template for the blank group. The unpaired *t* test was applied for the statistical analysis in GraphPad Prism v8. *, *P* < 0.05.

In this study, when the RPA reaction mixture was placed immediately into the CRISPR/Cas12a system, the Cas12a protein induced powerful nonspecific single-stranded DNA (ssDNA) transcleavage through specific CRISPR RNA (crRNA)-guided cleavage of double-stranded DNA (dsDNA) (2), which can be collected and analyzed by lateral flow strips or a portable fluorescence thermostatic amplifier (3) (Fig. 1A).

We chose the F3L and B6R genes as targets for detection. Based on the newly published MPXV sequence (GenBank accession number ON563414.3), we designed specific crRNAs and found that they could be used to distinguish MPXV from variola viruses (VARVs) (see Fig. S1A and B in the supplemental material). To further test the specificity of the system, we also synthesized homologous fragments of other orthopoxviruses, including VARV E3L and B7R (GenBank accession number NC_001611.1), cowpox virus (CPXV) F3L and B4R (GenBank accession number X94355.2), and vaccinia virus (VACV) E3L and B5R (GenBank accession number LT966077.1). The detection system could not identify and amplify the target genes, therefore, Cas12a does not generate a cleavage signal, leading to negative results (Fig. 1B and C).

Subsequently, we identified the limit of detection (LOD) for MPXV F3L as 10 copies/μL and that for B6R as 1 copy/μL (Fig. 1D). Compared with the real-time PCR (see Fig. S1C and D), our system improves detection sensitivity by a factor of 10.

To test the ability of the system to cope with clinical samples, pseudotyped MPXVs containing the F3L and B6R fragments were obtained using Ad5 replication-defective adenovirus and diluted with throat swab and serum specimens. We obtained the same experimental results as described above (Fig. 1E and F). The lateral flow strip assay showed a sensitivity similar to that of the fluorescence thermostatic amplifier (Fig. 1G), and the test results were quantified (Fig. 1H).

Recently, we read with great interest the article by Lu et al. (4), which combined the RPA and Cas12a complex in a one-step fluorescence assay. The assay adopted suboptimal PAMs (sPAMs) instead of canonical PAMs. Based on the research, we synthesized three crRNAs targeting sPAMs TTGG, TCTG, and ATTA (see Table S1). These crRNAs exhibited more sensitivity than TTTA in the one-pot reaction (see Fig. S1E). Because it had the shortest time of detection, TTGG was used for the following assay. Meanwhile, we designed a crRNA targeting the TTGG sequence of the B6R gene. The LOD reached 100 copies/μL (Fig. 1I and J). Although the sensitivity is not as good as that of the two-step method, the potential contamination risk is greatly reduced because there is no need to open the cover during the operation. There are at least three or more base mutations in other orthopoxviruses, resulting in negative signals (see Fig. S1F and G). In contrast, the crRNA we designed is highly conserved in the MPXV sequence, in both MPXV strains Zaire-96-I-16 (MPXV-ZAI) and MA001 (MPXV-USA) (see Fig. S1H and I).

In conclusion, in order to facilitate the accurate diagnosis of early suspected cases of infection and to detect samples rapidly and stably, we designed a technology combining RPA with CRISPR-Cas12a that can detect MPXV quickly and sensitively. The system can detect MPXV genomic DNA in 40 min, with an LOD of 1 copy/μL, resulting in a highly sensitive and low-cost tool for detection of MPXV.
