# Supplementary material for: CRISPR/Cas12a Technology Combined with Recombinase Polymerase Amplification for Rapid and Portable Monkeypox Virus Detection
Source: Microbiol Spectr. 2023 May 8;11(3):e04233-22. doi: 10.1128/spectrum.04233-22 (PMC10269670; doi:10.1128/spectrum.04233-22)
Supplement: Supplemental file 1 — Supplemental material. Download spectrum.04233-22-s0001.pdf, PDF file, 0.7 MB [file spectrum.04233-22-s0001.pdf]

Supplementary Materials

Declarations

Ethics approval and consent to participate

The studies involving human participants were reviewed and approved by Ethics Review Committee of Tianjin University, Tianjin University. The ethics committee waived the requirement of written informed consent for participation.

Consent for publication

Not applicable.

Availability of data and materials

The raw data supporting the conclusions of this article will be made available by the authors, without undue reservation.

Competing interests

The authors declare no competing interest.

Funding

This work was supported by the National Key Research and Development Program of China (2017YFA0205102) and the National Science Foundation of China (32170144).

Author contributions

TW conceived the study. FL, SL and MH designed the experiments. TW, YT, SL supervised the project. YT, FL and MH analyzed the data. FL, SL, BL and MH wrote the paper. All authors read and approved the final manuscript.

Acknowledgements

We thank Chinese CDC (Chinese Center for Disease Control and Prevention) for serum provision.

Materials and Methods

Nucleic acid preparation

The F3L (29763-31211) and B6R (165115-166068) gene fragment of MPXV (GenBank: ON563414.3) was synthesized by GENEWIZ (Suzhou, China) and cloned into the pUC57 vector. Concentration was determined with Nanodrop and stored at -80°C until use. The crRNAs and primers were also synthesized by GENEWIZ. The monkeypox virus-F3L pseudovirus(TSV4731) and monkeypox virus-B6R pseudovirus(TSV4724) were purchased by Tsingke Biotechnology(Beijing, China).

DNA extraction

The extraction method of viral nucleic acid in the simulated sample was as follows: The pseudovirus solution was diluted in the throat swab or serum sample to a final volume of 200 μL. Genomic DNA extraction kit (DP304-02) was used to extract DNA through the principle of silica gel membrane specific adsorption of nucleic acid, in accordance with the manufacturer’s instructions. The standard plasmid carrying pseudovirus specific gene was used to make the standard curve. The copy number of foreign genes carried by the pseudovirus can be obtained by detecting the Ct value of the extracted

pseudovirus genes.

CRISPR/Cas12a detection reaction

The isothermal amplification of the F3L gene and B6R gene was performed with a commercial recombinase polymerase amplification (RPA) kit (TwistAmp, cat. no. TABAS03KIT) in accordance with the manufacturer's instructions. Briefly, 8 μL DNA sample, 2.5 μL forward prime, 2.5 μL reverse primer (10 μmol/L), 29 μL Primer Free Rehydration buffer, 6 μL DEPC water, and 2 μL activator was incubated at 39°C for 20min. 4 μL crRNA (1 μmol/L) and 3 μL buffer was incubated with 1 μL Cas12a (1 μmol/L) (New England Biolabs, cat. no. M0653S) at 25°C for 20 min and fully combined. Then, a 20 μL RPA reaction system and 2 μL FQ ssDNA Reporter (10 μmol/L, labeled with FAM and BHQ1) were transferred to the CRISPR/Cas12a cleavage assay. Reactions were incubated in a GS8 Isothermal Cycler (GenDX, cat. no. GS8) for 20 min at 37°C, with fluorescence measurements taken every 30s.

CRISPR/Cas12a reaction was added into 25 μL RPA reaction system to detect the reaction and 2 μL ssDNA FB probe (100 μmol/L, labeled with FAM and biotin) was used for ICS. The above was added in 65 μL detection buffer, then the strips (Milenia Biotec, cat. no. MGDH1) were inserted and incubated at room temperature for 10 min. The strips were then removed and photographed with a camera. The band density was analyzed by ImageJ for visualization and quantification.

One-pot reaction

The reactions were performed in 30 μL reaction volumes containing 1 μL LbCas12a (1 μmol/L), 4 μL crRNA (1μmol/L), 5 μL FQ ssDNA Reporter (10 μmol/L), 1 μL dsDNA substrate and RPA components(18 μL) according to the manufacturer's instructions (Weifang Amp-Future Biotech: WLB8201KIT). And subsequently, 1 μL buffer B were supplied before readout through fluorescence thermostatic amplifier at 37°C. Since the prophase was mainly in the RPA stage of amplifying DNA template and the detection signal generated by cleavage was weak, we started collecting signals after 10min of incubation.

78 **Supplementary Figure**

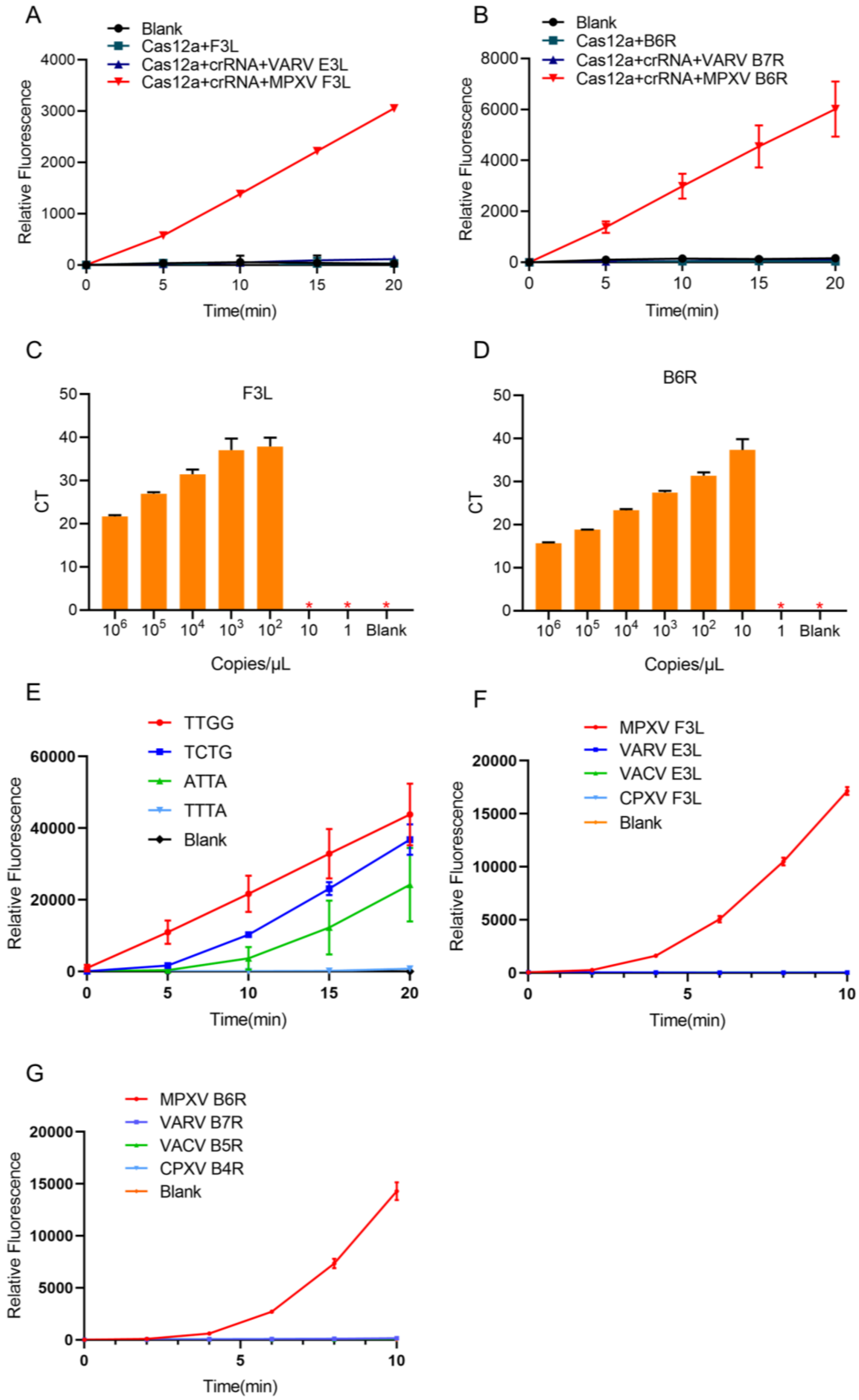

79

H

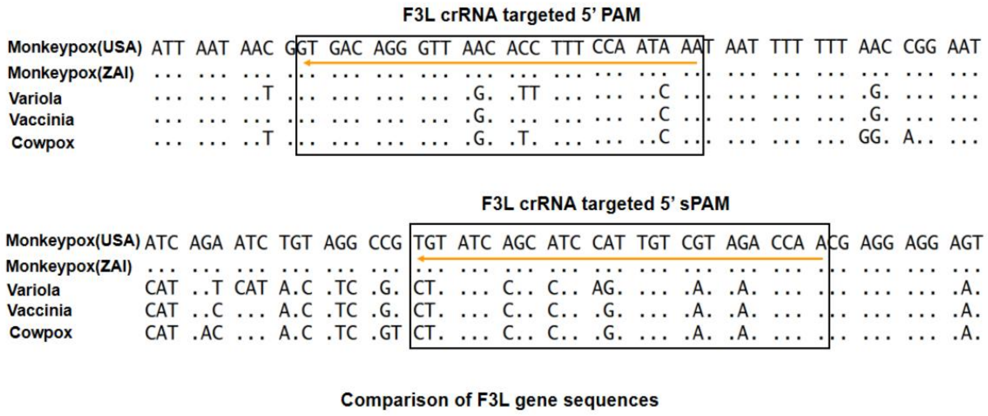

I

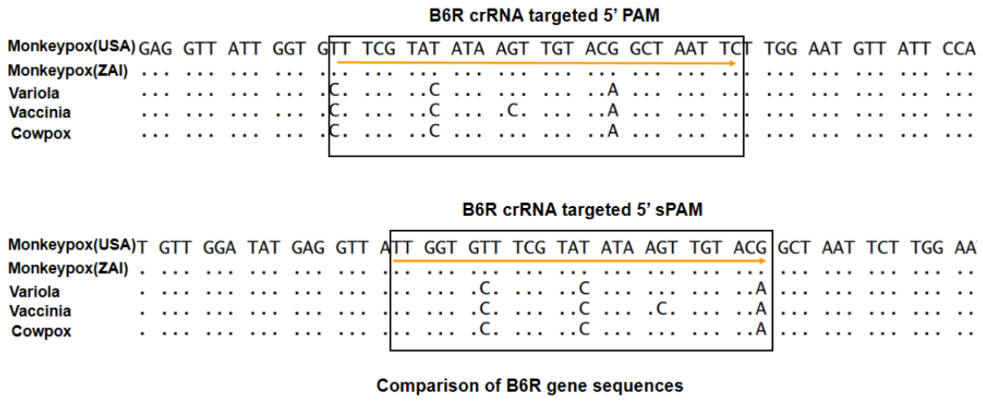

Fig. S1 A. To explore the specificity of crRNA, CRISPR/Cas12a system was used for detect the Cas12a+crRNA+MPXV F3L, Cas12a+crRNA+VARV E3L, Cas12a+F3L, and blank. **B.** CRISPR/Cas12a system was used for detect the Cas12a+crRNA+MPXV B6R, Cas12a+crRNA+VARV B7R, Cas12a+B6R, and blank. **C and D.** The gradient dilution of F3L and B6R genes were used as template for real-time PCR detection according to the instructions (Red * indicates no signal). **E.** Comparison of cleavage activity of Cas12a RNP complexes, which composed of different crRNAs using suboptimal PAMs TTGG, TCTG, ATTA and canonical PAM TTTA. The final concentration of F3L is 10^7 copies/μL. **F and G.** The DNA of monkeypox virus (MPXV) (10^5 copies/μL, final concentration, B6R and F3L gene), variola virus (VARV), vaccinia virus (VACV) and cowpox virus (CPXV) were

amplified by one-step fluorescence assay. **H and I.** The comparison of crRNA recognition sequences of F3L and B6R genes in MPXV, VARV, VACV and CPXV.

Table S1 List of primers, probe and crRNA

Name		Sequence
F3L	crRNA-TTTA	UAAUUUCUACUAAGUGUAGAUUUGGAAAGGUGUUAACCCUGUCAC
	Primer F	TAGGAGAGTTACTAGGCCCCAC
	Primer R	ACGACAATGGATGCTGATACAC
	crRNA-TTGG	UAAUUUCUACUAAGUGUAGAUUCUACGACAAUGGAUGCUGAUACA
	crRNA-TCTG	UAAUUUCUACUAAGUGUAGAUAUGCUGAUGCUAUAAUAGAUGAUG
	crRNA-ATTA	UAAUUUCUACUAAGUGUAGAUUAGCAUCAGCAUCAGAAUCUGUAG
	Primer F	ACTAGGCCCCACTGATTCAATA
	Primer R	TGATCTTCAACGTAGTGCTATG
B6R	crRNA-TTTC	UAAUUUCUACUAAGUGUAGAUGUAUAUAAGUUGUACGGCUAAUUC
	Primer F	CTAATGCGGAATGTCAACCTCTTCAA
	Primer R	AGAAAATGTAGATCCGGAAATTAATC
	crRNA-TTGG	UAAUUUCUACUAAGUGUAGAUUGUUUCGUAUAUAAGUUGUACGG
	Primer F	CTAATGCGGAATGTCAACCTCT
	Primer R	ATAACGCCACCGATAGAAAATG
F3L	Probe	5’FAM-CATCAGAATCTGTAGGCCGT-3’BHQ1
	Primer F	CTCATTGATTTTTCGCGGGATA
	Primer R	GACGATACTCCTCCTCGTTGGT

B6R	Probe	5'FAM-TCCCAACATGTGTACGATCTAACGAAGA-3'BHQ1
	Primer F	CGACGGTAAATGGAATCCCATAC
	Primer R	CGTCGGGACCATCATCCA
ssDNA-FQ reporters		IAB-TTATT-BHQ-1
ssDNA-FB reporters		FAM-TTATT-BIOTIN
